# Important Variation in Vibrational Properties of LiFePO_4_ and FePO_4_ Induced by Magnetism

**DOI:** 10.1038/srep33033

**Published:** 2016-09-08

**Authors:** Ali Seifitokaldani, Aïmen E. Gheribi, Anh Thu Phan, Patrice Chartrand, Mickaël Dollé

**Affiliations:** 1 LCES Laboratory of Chemistry and Electrochemistry of Solids, Department of Chemistry, Université de Montréal, P. O. 6128, Downtown Branch, Montréal, Québec, H3C 3J7, Canada; 2 CRCT Center for Research in Computational Thermochemistry, Department of Chemical Eng., Polytechnique Montréal (Campus of Université de Montréal), Box 6079, Station Downtown, Montréal, Québec H3C 3A7, Canada

## Abstract

A new thermodynamically self-consistent (TSC) method, based on the quasi-harmonic approximation (QHA), is used to obtain the Debye temperatures of LiFePO_4_ (LFP) and FePO_4_ (FP) from available experimental specific heat capacities for a wide temperature range. The calculated Debye temperatures show an interesting critical and peculiar behavior so that a steep increase in the Debye temperatures is observed by increasing the temperature. This critical behavior is fitted by the critical function and the adjusted critical temperatures are very close to the magnetic phase transition temperatures in LFP and FP. Hence, the critical behavior of the Debye temperatures is correlated with the magnetic phase transitions in these compounds. Our first-principle calculations support our conjecture that the change in electronic structures, i.e. electron density of state and electron localization function, and consequently the change in thermophysical properties due to the magnetic transition may be the reason for the observation of this peculiar behavior of the Debye temperatures.

Exhaustion of non-renewable energy resources is a major current concern. Among many others, the automobile industry is not exempt and a new means of transportation – electric cars – is being developed. However, at the moment, an electric car is still not affordable for most people, due to its high-priced battery. Lithium-ion batteries are the most popular types of rechargeable energy sources among others for electric vehicles. LFP – with olivine structure – was proposed by Padhi *et al.*^1^ in 1997 as a promising cathode material for high-power, safe and long-lasting Li-ion batteries; it continues to be the object of battery material research^2^. It very quickly became the center of attention for researchers and industries in energy contexts and opened the door for Li-ion batteries to take their place in large-scale applications, such as plug-in hybrid vehicles or electric vehicles^3^. This material displays several advantages over conventional cathodes: low cost, improved safety, and performance. Other attractive features are: high chemical stability, low toxicity, high theoretical reversible capacity (170 mAh/g) and an extremely flat charge-discharge profile at a reasonably high potential of ~3.4 V^4^,^5^.

In Li-ion batteries, LFP might be used as the cathode material while the anode is in general graphite. The cathode and anode materials are arranged in alternative layers and Li^+^ transfers through these layers. Charging and discharging is related to a reversible pumping of lithium ions from one electrode to another through reversible intercalation and de-intercalation processes. Many studies^6^,^7^,^8^,^9^,^10^,^11^ have shown the dependence of the open-circuit voltage (OCV) at low rates of charging and discharging on the FP–LFP phase diagram. The dependence of the electromotive force (emf) of LFP–Li ion batteries on chemical potentials, and consequently on the FP–LFP phase diagram, is well explained by Ichitsubo *et al.*^9^ and Xie *et al.*^11^. The electrochemical reaction of the LFP cathode can be expressed as follows:


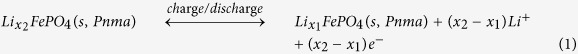


Here, *x*_1_ and *x*_2_ vary from 0 to 1 at ground state; however, this range is limited at higher temperatures due to the solid solution of LFP and FP. Considering the room temperature condition to use a battery, *x*_1_ and *x*_2_ are very close to 0 and 1, respectively, and the thermophysical properties of pure LFP and FP phases may be satisfactorily enough to treat the phase diagram. However, at higher temperatures the variation of *x*_1_ and *x*_2_ with temperature is more significant and cannot be neglected. The OCV depends on the chemical potential of lithium in a lithium metallic anode and on that in an olivine-type lithium phosphate cathode:


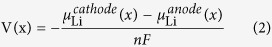


where *F* is Faraday’s constant; 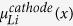 is the chemical potential of Li in the intercalation compounds; 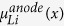 is the chemical potential of metallic Li and *n* is the charge (in electrons) transported by lithium through the electrolyte. The average voltage over the charge states between *x*_1_ and *x*_2_ can be written as^11^:


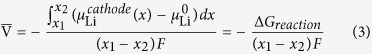


Here Δ*G* is the Gibbs energy change, where Δ*G* = Δ*H* − *T*Δ*S* here Δ*S* (entropy) and Δ*H* (enthalpy) are temperature dependent and are functions of the specific heat capacity (*C*_*p*_):


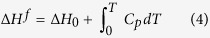



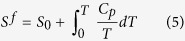


Hence, an accurate value of *C*_*p*_ is essential for accurate values of the Gibbs energy of the phases, and consequently for investigation of the FP-LFP phase diagram in understanding the battery operation. Currently, LFP is commercially available from mass production by different methods. Many improvements in materials and production processes have been achieved since it was first introduced. Nevertheless, our thermodynamic knowledge of FP and LFP is limited to a handful of experimental reports^12^,^13^,^14^ on heat capacity and a few computational studies^15^,^16^ to estimate elastic properties in the ground state. Enlarging our knowledge of thermophysical properties of FP and LFP, such as elastic properties, Debye temperature, and heat capacity at higher temperatures, is a crucial key to expanding available thermodynamic information, and consequently to answer questions about the phase equilibria in production processes.

In this article, we use a thermodynamically self-consistent (TSC) method to obtain the Debye temperatures of FP and LFP from available experimental specific heat capacities in a wide temperature range. These data are used to study the interesting and peculiar behavior of the Debye temperature as a function of temperature. This critical behavior is conjectured to be due to the magnetic phase transitions of both FP and LFP that change their electronic structures and consequently their mechanical and thermophysical properties. We used first-principle calculations to study electronic and crystalline structures of different magnetic phases of FP and LFP and consequently to understand this unusual behavior of the Debye temperature. The influence of magnetic ordering on vibration properties of transition metals compounds and oxides is already reported in literature^17^,^18^ (and the references within them). However, our approach in this study, to correlate the effects of magnetic ordering on the vibrational properties from a macroscopic perspective and through the study of the critical behaviour of the Debye temperature is very original.

## Methods

The Debye Temperature (*θ*_*D*_) is an essential parameter to connect thermophysical properties of solids to their elastic nature, through which one can calculate the heat capacity contribution of vibrational effects. Joardar *et al.*^19^ showed that the Debye temperature for orthorhombic crystals might be calculated as a function of the average sound velocity obtained by integrating the elastic wave velocities over 10 crystal directions ([100], [010], [001], [110], [011], [101], [√310], [1√30], [01√3], [10√3]) using the elastic constants as:


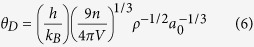


Here, *n*, *V*, *ρ*, *k*_*B*_ and *h* are the number of atoms in a unit cell, unit cell volume, density, Boltzmann and Planck constants, respectively. *a*_0_ is expressed as a function of 9 elastic constants 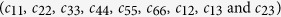 in ref. 19. To calculate the elastic constants of orthorhombic FP and LFP with *Pnma* space group, one needs to apply very small strains (to remain in the elastic limit of the crystal) to the equilibrium lattice (***R***) and to calculate the relevant total energies of the distorted lattices (***R′***) by density functional theory (DFT) calculations. Elastic constants (*c*_*ij*_) are proportional to the second order coefficient in a polynomial fit of the total energy as a function of the strain. The distorted lattice vectors for calculation of each elastic constant are obtained by multiplying the relevant distortion matrix (***D***) by the equilibrium lattice as described in detail in ref. 20 (***R′*** = ***RD***). Therefore, nine distortion matrices were used to obtain nine independent elastic constants of orthorhombic crystals. For each elastic constant, 11 strains in the elastic limit (less than 1% volume shrinkage or expansion) were applied to obtain a precise polynomial fitting. These calculations were repeated under external pressures (0, 1, 2, 3 and 4 GPa) to obtain the pressure and volume dependence of the elastic constants and also the Debye temperature. Hence, one could deduce the pressure derivative of the bulk modulus (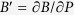) and also the first (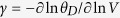) and second (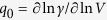) Grüneisen parameters.

The quasi-harmonic approximation (QHA) combined with the Debye model, may be used to calculate the vibrational contribution to the heat capacity at constant volume and different temperatures as:


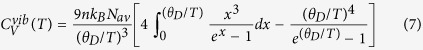


Here, *N*_*av*_ is Avogadro’s number. In addition to the harmonic contribution due to the vibrational contributions to the heat capacity, one may consider the electronic contribution 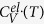, thermal vacancy defects 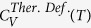 and magnetic effects 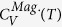 on heat capacity. However, because of a huge band gap observed in both FP (ca. 2.2 eV) and LFP (ca. 3.8 eV), electronic heat capacity is not significant. The contribution of thermal vacancy defects is apparent in available experimental data for the heat capacity at high temperatures close to the melting point. This contribution is normally represented by an Arrhenius-like function as:


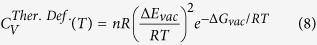


Here, *R* is the gas constant, Δ*E*_*vac*_ is the energy of formation of thermal vacancies, and 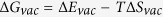
is defined with the energy (*E*_*vac*_) and entropy (*S*_*vac*_) of formation of thermal vacancies. Fitting the available experimental data to avoid the observed thermal vacancy effects at high temperatures below the melting point provides us with Δ*E*_*vac*_ ≈ 65 (kJ/mol) and Δ*S*_*vac*_ ≈ 30 (J/mol.K). The relevant concentration of vacancies (estimated by the concentration of monovacancies in a monatomic solid) may be estimated by^21^ 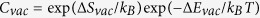, which gives us *C*_*vac*_ ≈ 0.21% and 5.5% at 800 and 1200 K, respectively. Although 5.5% vacancy around the melting point of LFP is theoretically possible, we believe that this amount is too big and we are probably neglecting other phenomena by abstracting them all in thermal vacancy defect. Following reaction shows that the exsolution of Li_3_PO_4_, that induces vacancy in Fe_3_P_2_O_8_, might affect the total heat capacity of the LFP. This happens at high temperatures. The exsolution and heat capacity of Li_3_PO_4_ is not considered in our rough estimation, which leads to the high thermal vacancy defects and consequently a high amount of concentration of vacancies at melting point.


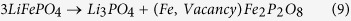


The magnetic contribution to the heat capacity is observed at low temperatures for both FP and LFP with magnetic transition temperatures 21~25 K^14^,^22^,^23^,^24^ and 50 K^12^,^25^,^26^,^27^, respectively.

In the QHA method, whatever success it may have, there lacks a systematic way of preserving the consistency of the properties and so conforming to thermodynamic laws. This deficiency has been recently eliminated through the TSC method^28^, which simultaneously conserves the thermodynamic laws. The TSC method is, in summary, an extension of the QHA satisfying the Maxwell relations to compel the results to conform to the fundamental thermodynamic laws, ensuring thermodynamic consistency. This method has been already examined for ionic oxides and metallic systems, and showed precise and consistent results more accurate than those from the classical QHA method^28^,^29^,^30^,^31^. The TSC iterative method, is based on the solution of a system of interrelated thermodynamic equations for the intrinsic properties of materials, in which the Debye temperature is one of the inputs provided from first-principle calculations. In this study, contrariwise, we obtained the Debye temperatures of FP and LFP in a wide temperature range below their melting points by fitting the available experimental data for the heat capacity with the TSC method. The Debye temperature varied from 100 to 1000 K. The obtained Debye temperatures are compared with the calculated Debye temperatures by ground state first-principle calculations for three different magnetic phases: ferromagnetic (FM), antiferromagnetic (AFM) and non-spin polarized (NS) materials. Therefore, through the TSC method, we are not only able to correlate the vibrational properties from a macroscopic perspective to the thermal properties, but also we preserve the thermodynamic consistency by eliminating the QHA model deficiencies. It is also worth to admit that, for having a microscopic understanding of the magnetic ordering effects on the vibrational properties the full phonon spectrum calculations and analysis is required.

The Vienna *ab initio* Simulation Package (VASP)^32^,^33^,^34^,^35^ has been used to perform the plane wave density functional theory (DFT) computations with the projected augmented wave (PAW) approach^36^,^37^ and the generalized gradient approximation (GGA) of Perdew, Burke and Ernzerhof (PBE)^38^,^39^. The Hubbard U correction to the GGA (GGA+U), equal to 5.3 eV for Fe in oxide systems, was employed to address a correct band structure and binding energies. Convergence in the energy and cell volume was tested and our results indicated that a cut-off energy of 520 eV and 3 × 4 × 5 Γ-centered k-points grid in the first Brillouin zone with a Gaussian smearing parameter *σ* of 0.02 eV ensure that the accuracy in the energy of the system is more than 0.01 meV. The self-consistent field (SCF) convergence criterion was 1 × 10^−5^ eV for electronic iteration and 0.02 eV/Å for each ionic loop that was updated by the conjugate gradient approach. To calculate the energy of the equilibrium lattice, the atomic positions, cell volume and cell shape, all were free to be relaxed. However, while applying a small strain to the system to calculate the energy of the system under stress, cell volume and cell shape were fixed and only the atoms were free to move. While calculating the electron density of state (DOS) and electron localization function (ELF), the k-points grid was 7 × 9 × 11 to achieve a finer band structure.

## Results and Discussions

The heat capacity of olivine-type LFP and FP have been measured in different calorimeters covering temperature ranges from 2 to 773 K^12^ and 2 to 300 K^14^ for LFP and FP, respectively. To the best of our knowledge, these data are the only reliable experimental data with specified accuracy. Loos *et al.*^12^ stated an error of 1 to 2% above 20 K and up to 8% below 20 K for LFP. They used three different calorimeters: the physical property measurement system (PPMS) from Quantum Design for the temperature range from 2 to 300 K, a Micro DSC II from Setaram^®^ within the range between 283 and 353 K, and a Sensys^®^ DSC from Setaram^®^ for the temperature range from 278 to 773 K. Shi *et al.*^14^ reported 1% error above 22 K and up to 5% below 22 K for FP. These heat capacity data are the basis of our TSC calculations for the Debye temperatures in a temperature range from 0 to 800 K.

In the TSC method, in addition to the Debye temperature at 0 K, we have to provide the values of the bulk modulus (*B*) – elastic constants (*c*_*ij*_) in general – and its pressure derivative (*B*′), molar volume (*V*) and also first and second Grüneisen parameters (*γ* and *q*_0_), all at ground state conditions. In the context of the TSC method, all these parameters are provided by first-principle calculations to predict mechanical and thermophysical properties at higher temperatures and pressures. However, in this study, we aimed to obtain a precise Debye temperature from experimental thermophysical data. We have used available experimental values for the molar volume (43.87 *cm*^3^*/mol* for LFP and 41.0033 *cm*^3^*/mol* for FP ref. 1). Our calculated molar volumes for FM and AFM are very close to the reported experimental values and are consistent with other first-principle studies^15^,^16^; Non-magnetic states, however, showed 3~4% lower molar volumes than the experimental values. For the bulk moduli, we have used the reported values of Maxisch *et al.*^15^ (93.9 GPa for LFP and 73.6 GPa for FP) and Shang *et al.*^16^ (89.5 GPa for LFP). Changes in the predicted heat capacity due to the different values of the bulk modulus between these two amounts were not significant and our TSC calculations are based on bulk moduli of 90 GPa for LFP and 70 GPa for FP. Our calculated *B*′ (4.3 for LFP and 4.2 for FP) is consistent with the Shang *et al.*^16^ DFT computations (4.33 for AFM LFP and 4.37 for FM LFP). The TSC method was not sensitive to the variation of *B*′ up to 15%, and we have used our calculated values in this article. To the best of our knowledge there is neither experimental nor first-principle calculations data for the first and second Gruneisen parameters for LFP and FP. Therefore, we have used our DFT computations results (*γ* ≈ 1.4 and 1.2, and *q*_0_ ≈ 0.5 and 0.6 for LFP and FP, respectively). 2970 DFT computations (495 computations for each system) have been done to calculate these parameters.

**Figure 1:**
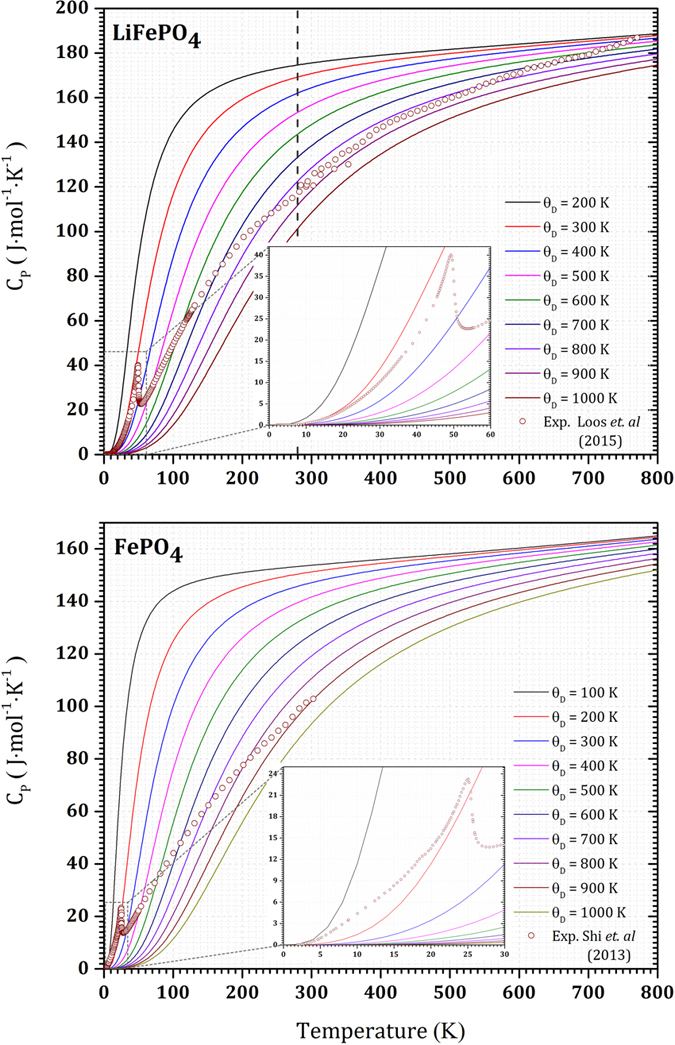
Experimental specific heat capacity (circles) and the predicted heat capacities by the TSC method for different Debye temperatures (solid lines). The vertical dashed line in LFP (also in Figs 2 and 3) indicates using of two different calorimeters bellow and above 280 K.

**Figure 2:**
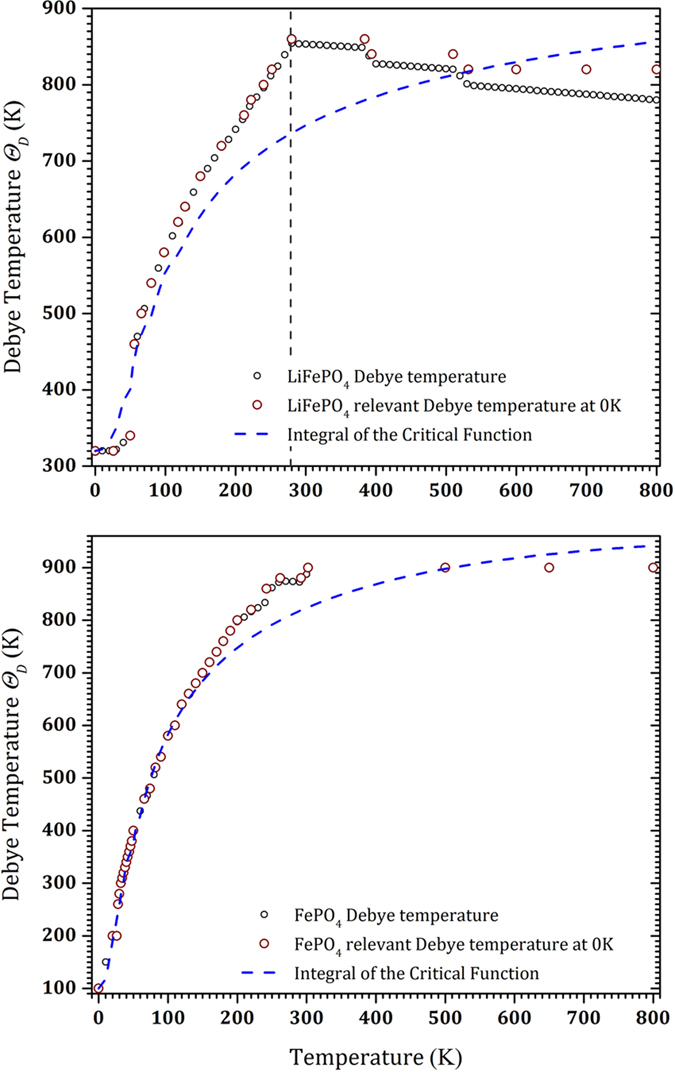
Calculated Debye temperatures from the experimental heat capacities by the TSC method at different temperatures (black circles) and their relevant amounts at 0 K (red circles). There is no experimental specific heat capacity data for FP at temperatures higher than 300 K. The data above 300 K in FP are, therefore, imagined to represent the heat capacity at these temperatures in analog to LFP. The blue dashed line represents the calculated Debye temperature by integrating the fitted critical function to the temperature derivative of the extracted Debye temperatures by the TSC method.

**Figure 3:**
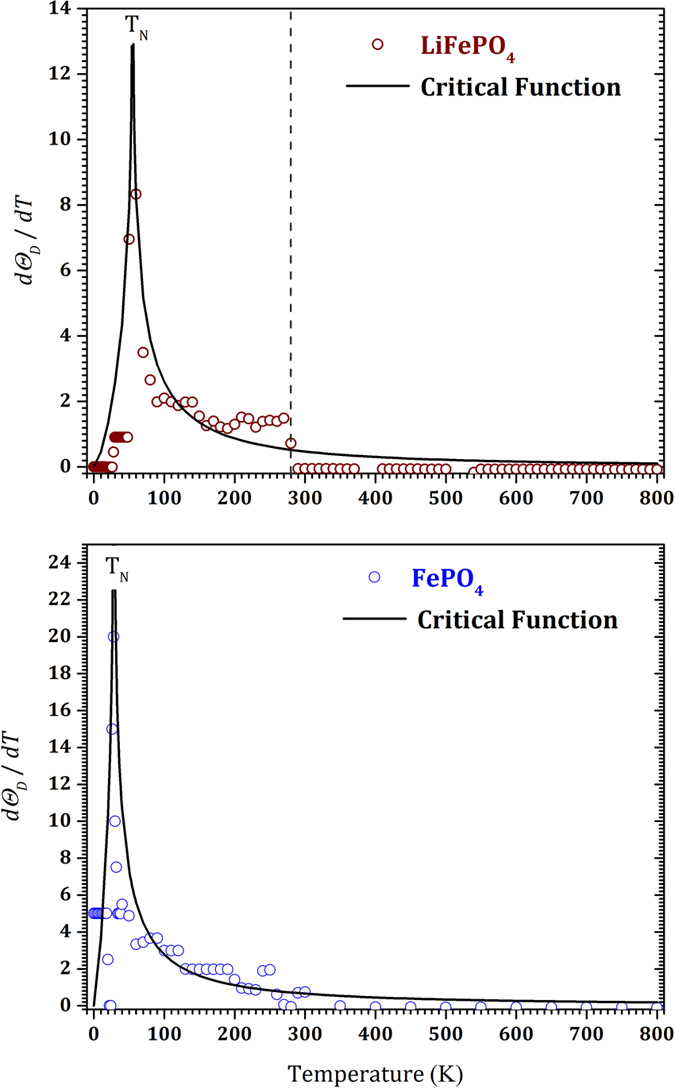
Temperature derivative of the obtained Debye temperature by the TSC method (circles). The solid line shows the fitted critical function.

Figure 1 depicts the experimental data along with a set of predicted heat capacities with various Debye temperatures from 100 to 1000 K using the TSC method. For non-magnetic materials, it has been shown that a single Debye temperature – in the ground state – might be enough to explain the thermophysical properties, including the heat capacity in a wide temperature range below the melting point^29^,^30^. However, for magnetic FP and LFP, we saw that one Debye temperature is not enough to cover a wide temperature range. Thus, at low temperatures we obtained a low Debye temperature and conversely at higher temperatures we obtained a greater Debye temperature to fit the experimental heat capacity. This behavior is already unexpected since we generally project an almost linear decrease of the Debye temperature at increasing temperatures. Figure 2 shows the Debye temperatures from which the predicted heat capacities fit the experimental data for both LFP and FP. At each temperature the Debye temperature and its relevant value in the ground state is shown. For LFP at temperatures higher than 600 K, experimental data show the thermal vacancy defects contribution. We fitted this region with the Arrhenius-like function in equation (8), in order to exclude the thermal defects contribution in the heat capacity. Hence, the calculated Debye temperature explains only the harmonic effects contribution to the heat capacity that remains almost constant around 800 K. In this figure, a break in the Debye temperature appears around 280 K. This break might be due to the using of two different apparatus in the heat capacity measurement bellow and above this temperature. A vertical dashed line at 280 K emphasizes this in Figs 1, 2, 3 for the LFP. However, the main transition is happened bellow this temperature and this issue does not affect our discussion in this paper. Unfortunately for FP, there are no experimental data above 300 K. In this study, therefore, according to similar behavior of the Debye temperatures that we observed for FP and LFP up to 300 K, we assumed that the Debye temperature for FP also remains almost constant around 870 K or with a small decrease above 300 K. Figure 3 clearly demonstrates the critical behavior of the temperature derivative of the Debye temperature for both FP and LFP. This critical behavior is fitted with the following model given in refs 40, 41, 42, 43:


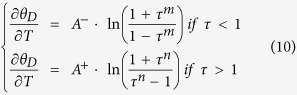


where, *τ* = *T*/*T*_*c*_ and *A*^−^, *A*^+^, m and n are adjustable parameters and *T*_*c*_ is the critical temperature. Although equation (9) was originally proposed to describe the second-order magnetic phase transition^40^,^41^, a similar function was employed to describe other second-order phase transitions, such as the porosity induced second-order phase transition in the thermal conductivity of sintered metals^42^,^43^. In Fig. 2 the integral of the fitted critical function – that is the Debye temperature – is shown. This function qualitatively justifies satisfactorily the sudden step-like increase of the Debye temperatures. The fitted critical temperatures are 55 and 28.5 K for LFP and FP, respectively, while the critical exponents are (m ≈ n) 1.5 and 1.3. These critical values are surprisingly in good agreement with the Néel temperatures of LFP (50 K)^12^,^25^,^26^,^27^ and FP (21~25 K)^14^,^22^,^23^,^24^, respectively. Other functions, i.e. critical exponent 
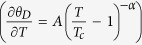
 and step-like functions such as Fermi-Dirac function 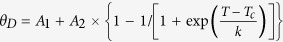 also were used to fit the Debye temperature and its temperature derivative. The fitted critical temperatures in these functions were very close to those obtained from the model in equation (9). Logically, we suppose that the transition in the Debye temperature might be due effectively to a magnetic transition that changes the electronic structure and consequently harmonic properties of the crystals. In previous works, Loos *et al.*^12^ and Shi *et al.*^14^ tried to fit the experimental heat capacities in the transition region with a function including both the Debye and Einstein temperatures simultaneously. Interestingly, the reported fitted values for the Debye and Einstein temperatures by these authors are very close to the two high and low limits of our calculated Debye temperatures, i.e. 235 and 930 K for LFP, and 131 and 683 K for FP. However, we believe that combining these two fundamental temperatures in one merely mathematical function has no physical meaning and is impractical.

**Figure 4:**
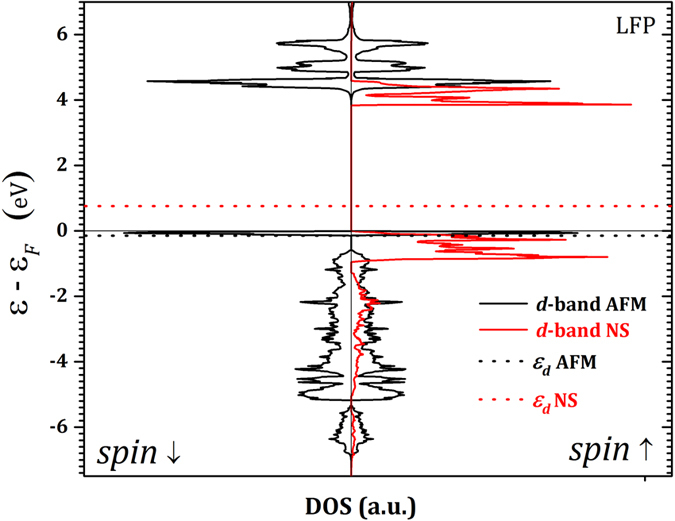
Projected *d*-band density of state (DOS) for AFM (black line) and NS (red line) LFP. The dot lines show the relevant calculated *d*-band centers (*ε*_*d*_).

**Figure 5:**
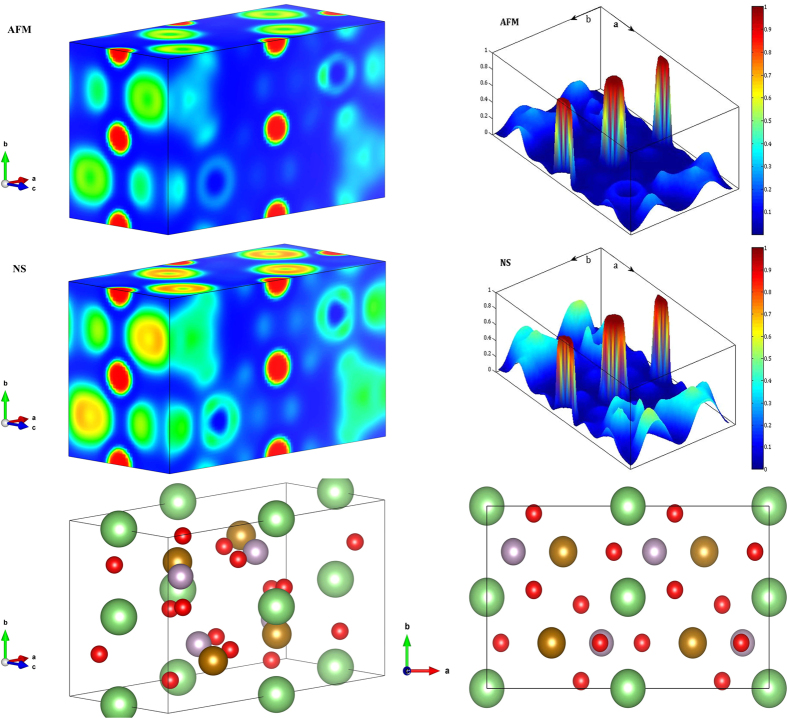
The 3D ELF of AFM (top-left) and NS (middle-left) LFP. The intensity of the ELF in ab surface is demonstrated for AFM (top-right) and NS (middle-right). In the bottom, we see the atomic positions in LFP unit cell. Green spheres stand for Li, red for O, pale violet for P and brown for Fe.

Our first-principle calculations prove that the magnetic phase transition alters the electronic structure and consequently the physical properties of both FP and LFP. The calculated Debye temperatures from the DFT computations, for magnetic (375 K for LFP and 294 K for FP) and non-magnetic (668 K for LFP and 649 K for FP) phases, show reasonable accordance with the extracted Debye temperatures from the experimental data at low and high temperatures, respectively. Electronic structure analysis showed that, despite a very similar charge density distribution for both magnetic and non-magnetic systems, there is a considerable difference in the *d*-band states between AFM and NS phases. Figure 4 shows that the *d*-band center increases from −0.15 eV below the Fermi level in AFM LFP to 0.76 eV above the Fermi level in NS LFP. Consequently, *d*-band width decreases from 4.46 to 3.20 eV from AFM to NS systems, respectively. This might affect the interaction between the iron 3*d* orbital and phosphorus and oxygen *2p* orbitals. The visualized ELF in Fig. 5 shows the different electron localizations in AFM and NS LFP. The ELF is a very powerful tool to categorize and evaluate the chemical bonding between elements in a molecule. In general, this function has a value between 0 and 1. Regions close to unity (red area) contain many localized electrons, which are either localized around a nucleus or in a very strong covalent bond. Values close to zero (blue area) show the region with low electron density and the value close to 0.5 (green area) represents a homogeneous electron gas where the bonding might have metallic character. In both magnetic and non-magnetic LFPs we see that lithium electrons are much localized around the Li nucleus, with a minor contribution making chemical bonding with other elements. However, the interactions between Fe and P and also between Fe and O are much stronger in NS LFP than those in AFM LFP. These stronger interactions are evident in interstitial regions between Fe, P and O with a greater electron density. In fact, in NS LFP, Fe tends more to form a hybridized *p-d* orbital through sharing its *d*-band electrons with *p*-band electrons of P and O. Due to the stronger interactions in NS LFP, we see that the Fe-O average bond length decreases from ca. 2.26 Å in AFM to 2.09 Å in NS LFP. In addition, the Fe-P average bond length decreases from 2.87 Å in AFM to 2.69 Å in NS LFP. These results, which are in complete agreement with our TSC calculations, are consistent with the greater bulk modulus and Debye temperature in NS LFP compared to AFM LFP. We have obtained similar results for FP; however, to avoid repetition we omit them here.

From the physical point of view, the third-order phase transition highlighted in this work shows the strong impact on the magnetic short-range ordering on the lattice properties in a wide temperature range above the Néel temperature. The Debye temperature behavior (Fig. 2) is associated with a smooth attenuation of the magnon-phonon contribution to heat transport in the critical region. For FP and LFP, the critical region is extended up to ca. 300 K, where the Debye temperature reaches a plateau. This temperature is the paramagnetic limit. The strong magnon-phonon interaction in a wide temperature range above the Curie temperature was recently explained by Kӧrmann *et al.*^44^ for bcc iron and a sketch for the evolution of the magnitude of the magnon-phonon contribution to overall lattice properties, in particular to the phonon frequency for different modes was proposed. Their model matches well with neutron scattering data. In our case, only a set of heat capacity data is available for each studied compound. Further, should be necessary to understand the mechanism of the magnon-phonon contribution to the lattice properties of FP and LFP.

## Conclusion

The Debye temperatures of FP and LFP have been calculated from experimental specific heat capacity data by the TSC method. These calculated Debye temperatures showed critical and unusual behavior, showing a sharp increase of the Debye temperatures with increasing temperature. This critical behavior was fitted by a critical function with a critical temperature close to the Néel temperature. First-principle calculations showed a significant difference in *d*-band states of these magnetic compounds before (magnetic) and after (non-magnetic) phase transition; thus, the non-magnetic state (high temperatures) had a *d*-band center greater than that in the magnetic state (low temperatures). In addition, ELF analysis showed stronger Fe-P and Fe-O bonding in non-magnetic compounds compared to the magnetic compounds. Therefore, shorter bond lengths and consequently greater bulk moduli and Debye temperatures were observed in non-magnetic systems compared to the magnetic systems. The calculated Debye temperatures through first-principle calculations for these two magnetic phases were in reasonable agreement with the extracted Debye temperatures by the TSC method at low and high temperatures. Hence, the critical behavior of the Debye temperatures was correlated with the magnetic phase transitions in these compounds that change the electronic structures and thermophysical properties.

## Additional Information

**How to cite this article**: Seifitokaldani, A. *et al.* Important Variation in Vibrational Properties of LiFePO_4_ and FePO_4_ Induced by Magnetism. *Sci. Rep.* **6**, 33033; doi: 10.1038/srep33033 (2016).
